# The physiology and psychophysics of the color-form relationship: a review

**DOI:** 10.3389/fpsyg.2015.01407

**Published:** 2015-11-03

**Authors:** Konstantinos Moutoussis

**Affiliations:** Department of History and Philosophy of Science, National and Kapodistrian University of AthensAthens, Greece

**Keywords:** color, form, vision, brain, physiology, psychophysics

## Abstract

The relationship between color and form has been a long standing issue in visual science. A picture of functional segregation and topographic clustering emerges from anatomical and electrophysiological studies in animals, as well as by brain imaging studies in human. However, one of the many roles of chromatic information is to support form perception, and in some cases it can do so in a way superior to achromatic (luminance) information. This occurs both at an early, contour-detection stage, as well as in late, higher stages involving spatial integration and the perception of global shapes. Pure chromatic contrast can also support several visual illusions related to form-perception. On the other hand, form seems a necessary prerequisite for the computation and assignment of color across space, and there are several respects in which the color of an object can be influenced by its form. Evidently, color and form are mutually dependent. Electrophysiological studies have revealed neurons in the visual brain able to signal contours determined by pure chromatic contrast, the spatial tuning of which is similar to that of neurons carrying luminance information. It seems that, especially at an early stage, form is processed by several, independent systems that interact with each other, each one having different tuning characteristics in color space. At later processing stages, mechanisms able to combine information coming from different sources emerge. A clear interaction between color and form is manifested by the fact that color-form contingencies can be observed in various perceptual phenomena such as adaptation aftereffects and illusions. Such an interaction suggests a possible early binding between these two attributes, something that has been verified by both electrophysiological and fMRI studies.

## On the Role of Color Vision

Color vision is an evolutionary gift to some organisms. As everything in nature has a purpose, it makes sense to ask what color vision is for, what type of purpose does it serve, and how does it improve the way in which visual perception supports knowledge acquisition about the world. In addition to esthetically enriching our visual experience, color vision has several more practical applications as well (for a review on the functions of color vision see Mollon, 1989). The most obvious one is that color gives information about vital things such as the state of ripeness of fruit, the suitability of flowers, as well as the presence of water (revealed by the color of the vegetation of a place). Furthermore, colors are also often used as sexual signals for reproduction, as well as indicators in estimating the health or emotional state of others. A major role of color vision, however, is to allow us to detect targets against dappled or variegated backgrounds, where lightness is varying randomly. Color is a linking feature of perceptual segregation serving the detection of targets, and thus the identification and categorisation of objects in the environment. But why should the brain bother to use chromatic input in analyzing shape, when much more detailed information is provided by the luminance system? Object segregation can also be performed by the latter, but color vision has the big advantage of being indifferent to local changes in illumination. Luminance and color edges usually occur together, but in the case of illumination edges, shadows, and highlights, the conservation of color supports the integrity of an object, making chromatic variations more reliable indicators of material boundaries. Thus, color vision helps segment a retinal image into perceived material and illumination components, which is critical for object perception (see Cavanagh, 1991; Shevell and Kingdom, 2008). Sensitivity to both luminance and chromatic contrast would be advantageous for an organism, providing redundant sources of information that would improve contour detection in noisy images. Furthermore, the information is not always redundant: a detailed study on the color-contrast and luminance-contrast statistics of natural images has shown that both variations occur equally often and are independent of each other (Hansen and Gegenfurtner, 2009).

Although the low-level characteristics of luminance-derived form vision are slightly better than those of the chromaticity-derived ones (see below), it has been demonstrated experimentally that the latter can sometimes do better than the former. For example, the strength and priority of color information in perceptual segregation is evident in a study in which color noise was shown to strongly interfere in an orientation-based texture-segregation task, rendering objects invisible to normal observers (Morgan et al., 1992). It is interesting that red-green color-blind dichromats escape the color camouflage and perform better than normal subjects in this task. Furthermore, it has been shown that even the S-cone signal alone can be used to detect chromatic boundaries in the absence of luminance contrast (Conway, 2014). It therefore seems that color information can support form perception in a way that can sometimes be superior to luminance information.

An important question that follows is whether these two different systems support form-perception independently, or whether and at what stage do they feed into a common mechanism. The fact that, in vision, we cannot concurrently entertain different perceptual organizations, might indicate a common pattern-recognition system (Mollon, 1989). On the other hand, color and form are two different attributes of visual experience, and it would make sense if they were processed separately by independent, functionally specialized systems. Such a functional segregation has been supported by the neurobiological architecture of the visual system (see Zeki and Shipp, 1988; Zeki, 1993), as well as by psychophysical studies showing that different visual attributes, including color and form, are being perceived independently and at different times (Moutoussis and Zeki, 1997a,b; Moutoussis, 2012). The question of separating chromatic from spatial vision is as old as Chisholm (1869) and will be the main topic of the present review.

## Early Separation of Brain Functions

Spectral variations in the environment are extracted by cone opponent mechanisms, which give rise to the chromatic system, whereas intensity variations are extracted by cone additive mechanisms, which give rise to the luminance system. The latter can use luminance contrast to support form perception (*form-from-luminance*). On the other hand, the variation in the wavelength composition of light reflected by the various parts of the visual field can serve two functions: the first is to be used in order to segregate and segment the external world into various objects (*form-from-color*), and the second is to assign to each of these objects a particular color (*color-from-color*). It therefore seems that we are equipped with two separate chromatic systems, one which is involved in the detection of edges and the analysis of spatial detail, and one which calculates the color of each object in the visual field (Mollon, 1989). It is obvious that the former system is able to support, and is directly involved with, form perception. However, the relationship between the second color system and the processing of form (whether luminance or chromaticity based) is also interesting, since they seem to be closely interlinked: a fundamental parameter of most color-computation algorithms is the spatial (and spectral) structure of the light entering the eye.

Land’s Retinex Theory of Color Vision is one such example. In this algorithm, the relative amounts of long-, middle- and short-wave light in each area of the visual field (a multi-colored ‘Mondrian’ was used in his demonstrations) is compared to the relative amounts of long-, middle-, and short-wave light in the other areas of the visual field, in order to calculate three ‘lightness-records’ for each area and thus determine its color (Land, 1974). Each ‘lightness-record’ is a calculation of the relative amount of the particular wavelength reflected from this area with respect to the amount of the same wavelength reflected by other areas in the scene – a spatial comparison. Before any such computation can be executed, the visual field has to be first segmented into different areas. It therefore seems that form perception, or at least form computation, is a necessary prerequisite for the calculation of perceived color.

The brain is equipped with neurons that could carry out such calculations (see below). An important distinction would be to ask whether a neuron is color-selective, in which case participation in color-calculations is more likely, or simply color-sensitive (signaling pure color-contours irrespective of the actual color), in which case a contribution to form-from-color is more probable. The distinction can be pictured as follows: those neurons that are interested in the spatial arrangement of the color-pair, reminiscent to simple cells in terms of receptive field construction, vs. those that are not, resembling complex cells in terms of receptive field construction. The terms *color-signed* and *color-unsigned* have been previously used for these two types of color-processing mechanisms (Dobkins and Albright, 1994), and will also be used in this review. At initial stages, both classes (signed and unsigned) of cortical neurons likely receive inputs from just one of the two retinogeniculate color channels (parvo or konio). However the distinction remains operative at subsequent stages, after cortical convergence of the parvo and konio channels, where unsigned neurons are capable of responding more universally to any chromatic contrast, whereas others are still selective for the spatial geometry of particular hues, i.e., retain the color-signed property – the signature for color-from-color processing.

The characteristics of color-related neurons are discussed in more detail in the section on electrophysiology. The bearing of color physiology upon color psychophysics (or vice versa) will be noted throughout the text, although this is far from cut-and-dried. As noted above, the guiding principle is that color sensitive (unsigned) cells should support form-from-color, and color selective (signed) cells support color-from-color. However, given the possibility that color signed signals at one stage may be pooled to form color unsigned signals at a higher stage, it is not possible to make the distinction unequivocally. For example, some neurons in area V1 reportedly combine color selectivity with sensitivity to luminance contrast, making the classification of these cells and their potential contribution to perception rather ambiguous. Thus, to be frank, the conceptual clarity offered by the use of the ‘signed/unsigned’ terminology is not intended to disguise the fact that the relationship between perception, psychophysics and hierarchical physiology remains a complicated (and unresolved) story.

## Form can Influence Color Perception

Several psychophysical studies have clearly demonstrated an interaction between color and form perception, with the latter being able to influence the former. Color filling-in experiments is one such phenomenon: in one of the oldest studies of this type, it has been shown that, if retinal stabilization is used to render a disk-annulus contour invisible, the color of the annulus fills-in to the central disk (which in reality has a different color) and makes it also disappear (Krauskopf, 1963). The perception of edges is thus a determinant of the extent of color assignment, and it has been shown that even illusory contours can determine the shape of an area to be filled-in by color: because the S-cone system has poor spatial resolution and is thus blind to edges (Mollon, 1995), yellow can be made to bleed inside a gray region of similar red-green excitation, until a luminance or illusory contour is met (Santana et al., 2011). Measurements of the time that it takes to fill-in the color of a region, argue in favor of independent determinations of the boundary of a chromatic region and of the color of that region (Santana et al., 2011). With transparent motion stimuli, color filling-in is determined by image segmentation and can occur simultaneously and independently at multiple different surfaces, even if these surfaces occupy the same retinotopic positions (Kanai et al., 2006).

It has also been shown that color induction, i.e., the effect of the color of the surround on the color appearance of a central target, is maximal at isoluminance, when there is no luminance contour between the center and the surround (Gordon and Shapley, 2006), suggesting that luminance can supress color just as color can suppress luminance (Alpern, 1964). Furthermore, color constancy calculations can be influenced by the segmentation of an image in 3D space, as separate constancy-computations seem to operate at separate depth planes (Werner, 2006). Another example on how form can determine the color of an object comes from the fact that the latter can be made to vary with the 3D perception of a surface: using goggles to change the appearance of a 3D corner between convex and concave will also change the color appearance of one side of this corner, depending on whether it is perceived as an inner or an outer surface (Bloj et al., 1999). Effects like these can be attributed to the fact that, in addition to on-line computations regarding the light composition reflected from various parts of the visual field, color calculations also take into account prior knowledge regarding factors such as the source and direction of the illumination. Bayesian inference, where the likelihood of a particular percept is not only determined by the current sensory data but also by the various priors of the system, has been extensively used in explaining color vision. A striking example of a cognitive influence in color perception is the demonstration that prior knowledge regarding the color of objects can make achromatic images to appear as colored (Hansen and Gegenfurtner, 2006a). In an elegant human fMRI study using pattern classification, the neural stamp of such priors was present as early as in area V1 (Bannert and Bartels, 2013). It thus seems that the mutual influence between form and color extends over all levels of hierarchical processing in the visual system, possibly using forward as well as backward pathways (Zeki and Shipp, 1988; Shipp et al., 2009).

## Color-Form Contingency and Double-Tuning

If color and form were processed in an independent manner, there should be no interaction between the two. More specifically, there should be a complete independence between the spatial characteristics of a stimulus, such as orientation or spatial frequency (SF), and its color. However, many psychophysical studies on illusions and perceptual effects resulting from *adaptation* (i.e., from changing the response of a system because of stimulation), reveal contingencies between these two visual attributes. The prevailing idea behind adaptation experiments is that neuronal populations selectively tuned to the adapting stimulus become fatigued after prolonged exposure to the latter, leading to a relatively higher sensitivity of opponent populations and thus to an imbalance of the system (see Kohn, 2007 as well as Thompson and Burr, 2009 for a review, including alternative explanations). Adaptation thus serves as the electrode of the psychophysicist in discovering neurophysiological properties of the brain: if a mechanism adapts, this is taken as an indication that it therefore must exist.

**FIGURE 1:**
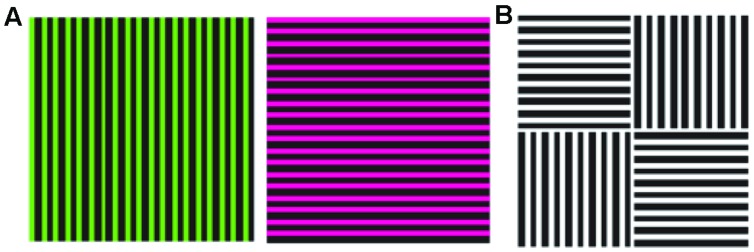
**The McCollough effect.** Alternative, retinotopically corresponding presentations of the two stimuli shown to the left **(A)** during an adaptation period, leads to orientation-specific color after effects: when the test stimulus shown to the right **(B)** is presented, the vertical bars appear red and the horizontal bar appear green (Reprinted with permission from Nature Publishing Group, Macmillan Publishers Ltd, Nature Neuroscience; Vul and MacLeod, 2006).

Probably the oldest and most widely known example of a contingent aftereffect is the *McCollough effect*, which is an orientation-specific color-aftereffect (see **Figure 1**): if one adapts simultaneously to two color-orientation pairs, the color of the color-aftereffect depends on the orientation of the test stimulus (McCollough, 1965). For example, if one takes two complementary colors such as red and green, and attributes them to a vertical and a horizontal grating respectively during an adaptation period, a vertical achromatic test stimulus will produce a color-aftereffect with a green tinge, and a horizontal achromatic test stimulus will produce a red tinged-aftereffect. Since the visual system has adapted equally to the two colors, the presence of a color-aftereffect suggests that color and orientation are encoded as a couple. The effect does not show interocular transfer, suggesting that it takes place early in vision – area V1 actually being the only candidate, since there is no orientation selectivity before and no monocularity after. The color specificity of the aftereffect suggests the involvement of striate neurons that are selective to both color and orientation. Interestingly, if the two adaptation pairs are presented at high alteration rates that make not only the color-orientation pairing but also the two colors themselves invisible, the aftereffect is still there, further suggesting that this early binding of color and orientation is also preconscious (Vul and MacLeod, 2006).

A similar but opposite contingency between color and form has been demonstrated using the *tilt-aftereffect*: double adaptation to two gratings of different colors, one tilted to the left and one tilted to the right, will make a vertical test grating appear tilted to the direction opposite to that of the adaptation grating of the same color (Held and Shattuck, 1971). The illusion is maximal at around 15° and then declines, in a way that permits one to calculate the width of the orientation tuning of the underlying mechanisms. It should be noted that, in both this and in the original McCollough studies, there was also a luminance contrast present – orientation was not defined purely by color. Thus, at the neuronal level, selectivity to color co-existed with selectivity to orientation which nevertheless was luminance-defined. However, it has been also shown that the normal tilt-aftereffect is equally strong using isoluminant stimuli, that there can be partial interaction between the luminance and the chromatic system in this effect, and that the effect is generally reduced as the difference between adaptation and test colors is increased, revealing in this way the tuning of orientation-specific mechanisms in color vision (Elsner, 1978; Lovegrove and Mapperson, 1981). Collectively, these results support the notion that both the luminance and the chromatic systems are equally efficient in signaling orientation.

**FIGURE 2:**
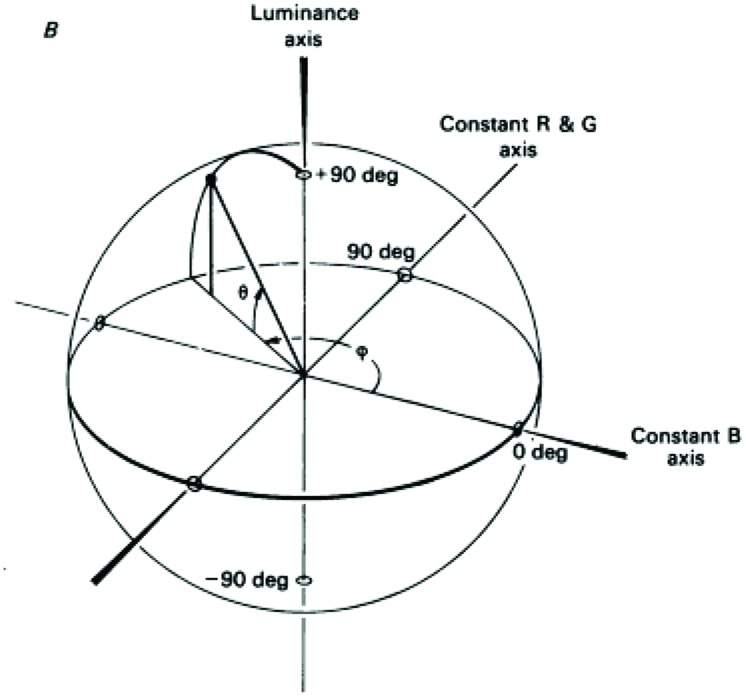
**A schematic demonstration of the DKL color space.** The ‘constant B’ and ‘constant R&G’ axes define an isoluminant plane centered on the white point. Each color is represented as a point in 3D space using spherical coordinates that specify the elevation from this isoluminant plane (i.e., modulation along the achromatic/luminance axes), the azimuth (i.e., the actual hue) and the contrast (as a fraction of the maximal modulation along the cardinal axes) (Reprinted with permission from John Wiley & Sons Ltd; Derrington et al., 1984).

In another detailed study for color-contingencies in the tilt-aftereffect it was found that, after double adaptation to oppositely oriented colored gratings, the direction of the aftereffect depended on the position of the test in color-space – being maximal when test and adaptor were identical (Flanagan et al., 1990). The color space used is explained in **Figure 2** and is the one defined by Derrington et al. (1984), also known as the DKL color space, the cardinal directions of which accurately describe the color preferences of parvo and konio LGN neurons (magenta/cyan and purple/greenish-yellow) rather than the primary perceptual opponent colors (red/green and yellow/blue). Color gratings were formed by sinusoidal modulations along the cardinal axes, and drifted during adaptation to prevent the formation of static afterimages. Adaptation along an axis 45° to the cardinal ones also gave a partial aftereffect, in accordance with physiological studies that report a broader distribution of preferred color axes amongst early cortical neurons compared to the LGN (Lennie et al., 1990; Kiper et al., 1997). As is also the case with the McCollough effect, cortical mechanisms must be involved in the tilt-aftereffect, since there is no orientation selectivity at a subcortical level. Concerning the color selectivity, the stimulus used by Flanagan et al. (1990) should adapt both color-signed and color-unsigned oriented cells; either class could be capable of generating the observed contingent aftereffect, the implication being that orientation tuning is generated separately within the parvo and konio channels at a cortical level, prior to their convergence. However, when only one color-orientation pair was presented during adaptation, the peak magnitude of the tilt aftereffect was larger, and never fell to zero (across test gratings of varying chromaticity, including achromatic). This suggests the presence of two components for the aftereffect, a baseline non-selective one and one which is tuned to color. The latter can encode form in a similar and equally efficient way to the former, providing strong evidence for the ability of the color system to support form perception independently. The baseline non-selective one, on the other hand, provides evidence for a second stage of form analysis, able to pool across chromatic and luminance inputs. Thus, the results of Flanagan et al. (1990) suggest the existence of several, separate orientation-tuned cortical mechanisms, one of which is achromatic and at least two of which are chromatic, and thus support the idea of different populations of orientation-selective neurons maximally activated by each. The existence of integrative systems at a higher level is supported by further evidence that is presented below.

Mechanisms responsible for the tilt-aftereffect are probably also responsible for the *tilt-illusion*, in which a tilted annular grating induces an opposite tilt in the orientation of a central target grating (Gibson and Radner, 1937). Unlike early reports claiming the failure of this illusion at isoluminance (Livingstone and Hubel, 1987), the tilt-illusion can be supported by purely chromatic stimuli and maximizes when the test and inducer are of similar chromaticity (Clifford et al., 2003a). This selectivity for color implies the involvement of color-selective cells, whereas the persistence of the effect using different colors reveals the involvement of neurons that are color-sensitive (but not selective) as well. Furthermore, an interaction between luminance and pure color gratings was also reported in this study, suggesting orientation-specific and color-specific lateral inhibition between cells preferring the same orientation. This, in turn, implies a dual selectivity and a color-form interaction at an early stage of visual processing (Clifford et al., 2003a). In a further study from the same group, comparing the tilt-illusion with the tilt-aftereffect, it was confirmed that both are maximal when test and inducer are modulated along the same axes of color space, with this selectivity being slightly more pronounced in the tilt-illusion (Clifford et al., 2003b).

The chromatic characteristics and the hierarchical position (within the visual pathway) of mechanisms involved in the tilt illusion were investigated in a study presenting the target to one eye and the surround to the other, with the two being either of the same or of different colors: it was found that, whereas the binocular mechanism is largely color-invariant, the monocular mechanism is purely chromatic, implying the existence of coupled color-orientation neurons in early visual cortex (Forte and Clifford, 2005). A two-stage model was suggested for the illusion, in which binocular visual mechanisms code for form in a manner that is largely insensitive to chromatic signature, whereas color and orientation processing interact at the monocular stages of visual processing (Forte and Clifford, 2005). Such a model is also in harmony with the lack of interocular transfer in the McCollough effect (McCollough, 1965). The existence of a form system based on pure chromatic information is further supported by experiments showing a decrease in threshold contrasts for vertical and horizontal orientations compared to oblique ones (*oblique-effect*), using isoluminant stimuli (Reisbeck and Gegenfurtner, 1998).

Using the *size-aftereffect* (Blakemore and Sutton, 1969), a contingency between SF and color has been also reported (Hardy and De Valois, 2002). In this experiment, subjects were adapted to colored, luminance-varying gabors, one with a high-SF and one with a low-SF, simultaneously presented at different retinal locations (see Figure 1 of Hardy and De Valois, 2002). If a single color (e.g., red) was used during adaptation, the aftereffect was stronger for a test of the same color but still present for a test of a different color (e.g., green). In double-adaptation experiments using two adaptors of a different color, also having the opposite spatial arrangement with respect to which SF was presented where, the direction of the aftereffect was found to depend on the color of the test (Hardy and De Valois, 2002). It was thus possible to dissociate between the color-selective and the color-insensitive mechanisms of the aftereffect, showing that the former could account for 1/3 of the total effect and was highly selective for both orientation and eye of origin, suggesting an early cortical monocular mechanism and the involvement of color-signed neurons (as in the McCollough effect). The color-insensitive part of the effect showed interocular transfer, revealing a later (higher) mechanism, and was (surprisingly) not so sensitive to orientation.

## Spatial Properties of Color and Luminance Detectors

As mentioned before, cone outputs are combined at the post-receptor level in two different ways: one additive, giving rise to luminance signals with no information regarding the wavelength composition of light, and one subtractive, which preserves the latter and can thus be used for determining the color of objects. An important question for visual-psychophysics is to examine the properties and functions of these two separate systems, as well as the relationship between them. According to the old ‘coloring book’ model, the role of the luminance system is to segment the visual input into different objects and shapes, and the role of the color system is simply to fill in the colors of these objects. An alternative view suggests that chromatic information can also be used to derive form perception, independent from and equally efficient to the luminance system. As we are not blind to stationary *isoluminant* (i.e., of the same luminance) figure-ground spatial arrangements, chromatic information seems capable of supporting the perception of form. The question is how well it can do this, and how its performance compares to that of the luminance system. For example, the spatial resolution of the latter is expected to be much better, since it is only limited by the spacing of (any type) of cones. Studies comparing the performance of the two systems with respect to spatial vision, as well as the degree to which they are independent or interact with one another, have shed some light on these matters. Detection thresholds, subthreshold (i.e., with intensities below threshold) summation, adaptation-aftereffects and masking, are the most common psychophysical tools that have been used in this quest.

One way to compare the properties of the color and luminance systems is to estimate their sensitivity by plotting *detection thresholds* as a function of *contrast*. In this way, the minimum contrast necessary in order to perceive the stimulus is estimated, and gives an idea of the sensitivity of the system to the particular attribute. There is a technical issue, however, when comparing the sensitivities of the color and luminance system, as the definition of contrast is not as straightforward in the former as it is in the latter. Luminance is what is called a *prothetic* continuum (‘how much?’), and thus has a measurable intensity (total amount of energy) that can be used in contrast calculations (e.g., [max-min]/[max+min]). Color, on the other hand, is a *metathetic* continuum (‘what kind?’), having no measurable intensity. Different studies have used different methods to define color contrast, usually in terms of graphics display capabilities, or with respect to an individual subject’s absolute detection thresholds. Such arbitrary definitions are useful in calculating things such as the sensitivity of the chromatic system with respect to SF, but leave the validity of a direct comparison between color and luminance performance an open question.

Along these lines, it has been shown that pure-color form is lost at high frequencies (see Elsner, 1978) and that, in general, the luminance system is considered to have a higher spatial (and temporal) resolution than the chromatic one (Kelly, 1983). The contrast-sensitivity function for color has been found to decrease monotonically with frequency, whereas the luminance one has drops at both the high and the low spatial frequencies (De Valois and Switkes, 1983; Mullen, 1985). When comparing the sensitivity of the two systems with respect to SF and orientation, luminance mechanisms show slightly lower contrast-detection thresholds, compared to color mechanisms (Webster et al., 1990). Such a comparison is made by normalizing luminance and color thresholds in terms of equal multiples of their respective contrast detection thresholds. Two types of tasks, across variable contrasts, were used by Webster et al. (1990): a detection task, in which subjects had to decide in which of two time intervals the stimulus appeared, and a discrimination task, in which subjects had to decide which type of grating was presented. It was shown that observers could reliably discriminate the orientation and SF of chromatic gratings even at the limit of the detection thresholds. Furthermore, they could also discriminate between luminance and chromatic gratings, as well as between different types of chromatic gratings at their detection contrasts, suggesting that the mechanisms involved in the detection of these stimuli are selective for both spatial and chromatic information.

Another way to examine the independence between different detection mechanisms in the brain is adaptation. Using adaptation-stimuli that isolate the S-cones, threshold-elevations that were specific to both orientation and color have been reported (Stromeyer et al., 1980). Results from this study are a clear demonstration of color-selective spatial mechanisms, as threshold elevation was not observed for colors others than the test. The effect did not show interocular transfer and was eliminated if either the test color or the test orientation were different from those of the adapting stimulus, revealing a combined orientation-color signal at an early stage of processing. Such evidence for a monocular color-orientation signal has been also suggested by other studies (see both above and below). With respect to SF, simultaneous but opposite size-aftereffects have been demonstrated after double adaptation to luminance and isoluminant gratings, suggesting that chrominance and luminance channels perform similar but independent SF analysis of the image (Favreau and Cavanagh, 1981). No interocular transfer was found for the isoluminant signal in this study, thus pointing toward the operation of early-stage mechanisms. Orientational anisotropy has been reported for the detection of chromatic gratings, also suggesting an explicit representation of the orientation of chromatic stimuli (Murasugi and Cavanagh, 1988). Furthermore, adapting to isoluminant gratings produced contrast-threshold elevations that were orientation and SF specific, just as is the case with adaptation to luminance gratings (Bradley et al., 1988). In this study, there was little cross-adaptation between luminance and color, pointing toward separate cell populations rather than a common form-system deriving input from both. Furthermore, the specificity with respect to the color of the stimulus might suggest the involvement of color-selective neurons.

The independence and interaction between different systems can be also revealed by the extent to which one can *mask* the other. In these types of experiments, the presence of a masking stimulus (usually referred to as *mask*) interferes with the detection of a target stimulus (usually referred to as *test*) presented closely in time. The mask can be presented slightly before, after, or at the same time with the test and usually results in increasing detection thresholds (although facilitation can occur with subthreshold masks). The idea is that if the mask has an effect on detection, then the two must be processed either by the same underlying neuronal mechanism or by different mechanisms that nevertheless interact with each other. If, on the other hand, the mask has no effect, it is assumed that the two are processed by independent mechanisms in the brain. For example, if masking effects are present only when test and mask have the same SF, an architecture characterized by separate spatial-frequency channels is implied.

Masking using luminance and isoluminant gratings has revealed a similar specificity to SF, suggesting that the color system, just like the luminance system (Campbell and Robson, 1968), also consists of bandpass (i.e., being of a specific frequency range) spatial filters (De Valois and Switkes, 1983). Within a system, spatial-frequency specificity was more pronounced for the luminance than for the color system, suggesting the existence of more broadly tuned spatial-frequency channels for the latter. Spatial-frequency specificity was even more pronounced when masking across systems, with the masking of color by luminance being the most stringent (identical spatial-frequencies required). This asymmetry indicates the possibility of asymmetric lateral inhibitory interactions at the neuronal level, with chromatic information dominating luminance information (De Valois and Switkes, 1983). These results clearly demonstrate that the two systems do not show complete independence and are extended in a subsequent study from the same group (Switkes et al., 1988), in which the mask was presented at subthreshold contrasts (suprathreshold masks were used in De Valois and Switkes, 1983). It has been discussed earlier (see above) that, due to shadowing, chromatic contrast is more reliable than luminance contrast in figure-ground segregation, and thus such an asymmetric interaction could be beneficial for the organism. Yet another masking study, using isoluminant stimuli of various spatial frequencies, has also verified that the chromatic contrast sensitivity function is the upper envelope of a range of bandpass mechanisms (Losada and Mullen, 1994). Close spatial-frequencies were again found to be more effective in both facilitation and masking than spatial-frequencies further away from the test. Therefore, as is the case with the luminance system, the color system also consists of different spatial-frequency channels but with a slightly broader tuning than luminance.

The sensitivity of the visual system is not always measured with respect to the total absence of a stimulus as a reference point, but also in cases where an initial stimulus (*pedestal*) is present. A subthreshold pedestal can either lead to facilitation of target detection or interfere with the latter, as in masking. Experiments showing that a color pedestal does not add to a luminance pedestal in facilitating luminance detection but produces masking instead (and vice versa), also point toward the existence of separate color and luminance spatial-mechanisms (Mullen and Losada, 1994). In the same study, subjects showed a decrease in test threshold with added luminance contrast (luminance-luminance facilitation), despite the initial threshold elevation (color-luminance masking) produced by a fixed chromatic pedestal. Subthreshold facilitation is thus unaffected by the presence of a suprathreshold color contrast mask, i.e., the opposite effects of masking and facilitation can occur simultaneously. These results can be explained by the presence of separate color and luminance mechanisms, which nevertheless interact with each other (Mullen and Losada, 1994). By testing subthreshold summation between color and luminance gabors over a wide range of spatio-temporal frequencies, the same group has failed to find any linear summation between color and luminance (Mullen et al., 1997). In this study, detection of the stimulus was achieved when either mechanism reached its own threshold (‘inclusive OR rule’), thus supporting the existence of two separate systems that contribute independently to detection using what is referred to as *probability summation* (but see also Gur and Akri, 1992). Similar results have been reported between all the possible combinations of the cardinal axes of the DKL color space (see **Figure 2**), normalized to detection threshold (Mullen and Sankeralli, 1999). In this study, subthresold summation revealed a stochastic independence of the ‘red–green,’ ‘blue–yellow,’ and luminance post-receptoral mechanisms, whose joint presentation at near threshold contrast raises the likelihood of detection through probability summation. It must be noted, however, that such results do not guarantee that independence is also present at suprathreshold levels (i.e., above threshold).

## Global-Form Computations

The detection and discrimination of local contours is the first stage in any computations leading to the perception of form. In the experiments described in the previous section, local contours were defined by luminance or color or both, and sensitivity to and interaction between luminance and chromatic stimulus characteristics were examined. Global form perception, however, is not fully characterized by the sensitivity and tuning characteristics of the different systems in the orientation and SF domain. It also requires an account on how the information of local detectors is integrated across space, and so one can ask whether chromatic and luminance local information are equally effective at this second stage, and whether these two systems remain separate or whether there are mechanisms able to pool local signals coming from different sources. Due to the possibility of such a pooling mechanism, a performance minimum at isoluminance in spatial-integration tasks does not necessarily imply a superiority of the luminance system, since it might also be the result of an additive mechanism to which both systems contribute (Kingdom et al., 1992). Such performance minima at isoluminance have been widely used to demonstrate a superiority of the luminance system in particular perceptual domains (e.g., motion – see Cavanagh et al., 1984). However, in order to infer a superiority of the luminance system over the color one, performance based on isoluminant and isochromatic stimuli requires direct comparison, raising the problem of calibrating their relative contrast, as noted above. This section describes experiments which are carefully designed to address the question of whether color and luminance information are equally effective in spatial-integration, and whether or not they combine at the level of global-form perception.

In one such study, in which low SF orientation pop-out was used in order to segregate figure from ground, after normalizing by the threshold for individual-element detection, no difference in the detection threshold of the figure was found between color-defined and luminance-defined gratings (McIlhagga et al., 1990). The results imply that such an automatic, pre-attentive texture-segregation process can be accomplished using chromatic information alone, just as efficiently as when using luminance information. Similarly, isoluminant stimuli were found to be equally effective to luminance ones in a spatial integration task, in which subjects had to judge the collinearity between 2 and 16 random elements (Kingdom et al., 1992). In yet another spatial integration task, it was shown that both luminance and chromatic stimuli employ probability summation of the orientation and contrast cues in order to detect a target within distractors – a process which also involves higher stages of global spatial processing (Reisbeck and Gegenfurtner, 1998). In this study, functions of orientation thresholds with respect to contrast were shown to have a similar shape for luminance and color, revealing similar orientation discrimination mechanisms (color being slightly more sensitive at low SF and vice versa at high SF). All these findings thus suggest that color contrast can be used as efficiently as luminance contrast in spatial integration tasks.

In another type of spatial task, in which the local orientation continuities of gabor elements are integrated into a global form pattern, the performance of the color system was found to be comparable to that of the luminance system, especially at high contrasts (McIlhagga and Mullen, 1996). When color-defined and luminance-defined gabors were combined, performance was much poorer compared to the homogeneous conditions, suggesting separate integration systems with a limited amount of interaction, rather than a single integration mechanism which is indifferent to the nature of the signal (McIlhagga and Mullen, 1996). In another contour integration task of this type, in which the element-to-element orientation curvature as well as the individual element contrast were the independent variables, performance was found to be similar if either luminance, or isoluminant ‘red/green,’ or ‘blue/yellow’ gabors were used as elements (Mullen et al., 2000). In this study, orientation discrimination was much weaker for the ‘blue/yellow’ stimuli. However, at threshold contrast for path detection all individual gabors were at suprathreshold contrasts, i.e., path detection performance was not limited by an inability to see the individual elements. It thus follows that the contrast limitation for this task must occur at a (possibly common) higher stage. Curvature, which was the other limiting factor for path detection in this study, was also found to affect the three types of stimuli in the same manner. So, although the optimal performances of the chromatic mechanisms fall slightly below that of the luminance mechanism, no evidence was found that they are deficient in contour integration. The authors also examined the possibility of a common integration process by using paths whose elements (gabors) alternated between two channels. This was found to impair performance, but not to the (very poor) level predicted by a model of performance using totally independent mechanisms (that would treat a 10-element contour as two separate five-element contours). Thus the spatial integration process responsible for path extraction was inferred to have some capacity to pool across the primary channels, but the nature of this process remained uncertain as it was not blind to the chromaticity of the gabor elements. Furthermore, it was also shown to be susceptible to variation in their phase, even with uniform chromaticity, suggesting some inherent involvement of color-signed mechanisms (Mullen et al., 2000).

In a subsequent study by the same group, the performance of the chromatic mechanisms was found to decline more steeply with increased element separation, suggesting that perhaps contour integration by this system relies more on short-range interactions compared to the more long-range interactions of the achromatic system (Beaudot and Mullen, 2003). In a different type of global-shape-discrimination study, in which radial-frequency was varied and subjects had to judge the circularity of a given pattern, the best performance was achieved with luminance stimuli and the worst with ‘blue/yellow’ (Mullen and Beaudot, 2002). Nevertheless, these differences were not very large and, at the highest contrast levels, chromatic shape discrimination could also reach hyperacuity performance, suggesting that color vision cannot be considered seriously deficient in global-form perception. Again, several different, color-orientation mechanisms seem to be involved, rather than simply a single chromatic and a single luminance channel.

A different spatial integration task, commonly used in the literature, is to measure the detection threshold of a *signal* which is embedded into *noise*, where signal and noise are sampled from separate Gaussian distributions across various directions in color space. It is namely a figure-ground segregation task based on the idea that, if noise and signal are being processed by separate neuronal mechanisms, modulations of the former should not interfere with the detection of the latter. If, one the other hand, changes in the noise affect the sensitivity to the signal, then a common underlying processing mechanism is assumed – i.e., the largest interference should be caused by noise modulations along directions close to the modulation of the signal. In this way, independent higher-order detection mechanisms can be identified, in addition to the cardinal ones: if noise orthogonal to one direction has no effect, this can be modeled by a higher-order, cortical mechanism which is tuned along this direction (see Figure 3 of Hansen and Gegenfurtner, 2013).

In a study measuring detection thresholds for vertical gratings embedded in spatiotemporal broadband noise, it was found that if signal and noise were modulated along orthogonal axes in a 2D color space (magenta-cyan and achromatic cardinal directions), the noise had no effect on detection (Gegenfurtner and Kiper, 1992). If, on the other hand, both noise and signal were modulated along the same axis, there was a linear relationship between noise and signal threshold. Furthermore, the slope of this relationship along the magenta-cyan and along the luminance axis was the same. These results suggest the existence of two equally efficient, independent mechanisms for the detection of chromatic and luminance signals respectively. Interestingly, other mechanisms tuned to non-cardinal directions that combine luminance and chromatic information have also been discovered, the chromatic tuning (preference and breadth of tuning) of which could be estimated by keeping the signal direction constant and changing the direction of the noise (Gegenfurtner and Kiper, 1992).

In another study along the same lines, minimal interference was found along cardinal directions, suggesting once more the existence of relatively independent luminance and chrominance channels, one in each cardinal direction, as well as another two channels in intermediate axes within the isoluminant plane (Li and Lennie, 1997). In yet another study of orientation discrimination involving external noise, performance was equivalent for color and luminance stimuli, demonstrating the ability of chromatic information to support the early stages of form vision, almost as effectively as luminance information (Beaudot and Mullen, 2005).

The question of the number of broadly-tuned independent chromatic mechanisms for form-segregation is of great interest, as it is clear that directions other than the cardinal ones exist in color space. In an attempt to investigate in detail the presence and number of such mechanisms, an image segmentation study used texture signal and noise that were varied across various directions in either the isoluminant or the L-M luminance plane, to show that masking is maximum when noise is in the same direction with the signal, and minimum when noise is in an orthogonal direction to the signal (Hansen and Gegenfurtner, 2006b). The tuning curves of detection threshold in color-space that resulted from these detailed measurements were best described by a chromatic-detection model of multiple (*N* = 16 gave the best fit) broadly-tuned, higher-order detection mechanisms. These results were verified by a subsequent study from the same group, in which higher-order color mechanisms were charted in a signal-within-noise setup, by keeping the noise constant and changing the direction of the signal (Hansen and Gegenfurtner, 2013).

Masking between color and luminance stimuli has also been used in higher-order form tasks, such as the detection of a gabor-defined, orientation-modulation pattern (see Figure 1 of Pearson and Kingdom, 2002). The idea is that if a subthreshold mask facilitates the detection of the test then both test and mask are processed by the same mechanism, something which was experimentally observed in both crossed and uncrossed conditions. Thus, orientation-modulation pattern-detection seems to be the function of a single mechanism pooling both luminance and color information. The authors propose the existence of different, independent color and luminance systems at the first stage of form-processing, and a common second stage system that pools color and luminance inputs (as proposed for contour integration in Glass-patterns – see below).

The potential for spatial integration of pure chromatic information with respect to global form perception has been also investigated using Glass-pattern stimuli (Glass, 1969). In these stimuli, a proportion of local elements (the signal) define a particular pattern (e.g., circularity) and the rest (the noise) are positioned randomly. Detection thresholds in such patterns were found to be highest when noise and signal had the same color, and to decline as the color difference between the two increased (Cardinal and Kiper, 2003). In this study, varying the distance in color space between isoluminant signal and noise in order to calculate tuning curves of threshold vs. color-difference, revealed the existence of several independent chromatic mechanisms, which are broadly tuned (i.e., sum their cone-inputs linearly). In another similar study (Wilson and Switkes, 2005), it was shown that chromatic information can be used to perceive form in Glass patterns, with detection thresholds similar to those of luminance (but slightly higher for the S-system). Additionally, by varying the color difference between the two elements forming a dot pair (‘intradipole’ variation) or between dot pairs (‘interdipole’ variation), the level of color-form integration could be measured at an early (local) or later (global) stage of processing respectively. Results showed that early-level integration is color-specific, i.e., the ability to extract the orientation of dot pairs in Glass patterns decreases with increasing chromaticity differences, whereas at a global (i.e., interdipole) level, the form system is able to integrate information from differing chromaticities, suggesting that later mechanisms responsible for pattern segregation are color-sensitive but not color-selective (Wilson and Switkes, 2005).

Glass-pattern experiments suggest that the local orientation of dot-pairs is calculated in the first stage, and in the second stage these local orientation signals are pooled over a large area of the visual field. By changing the color relationship between isoluminant dot pairs, it has been shown that first stage mechanisms are color selective and narrowly tuned in color space, i.e., do not combine their cone inputs linearly (Mandelli and Kiper, 2005). It thus seems that color selectivity is more pronounced at the early stages of form processing (Mandelli and Kiper, 2005), but still present at latter stages as well (Cardinal and Kiper, 2003). Adaptation experiments using radial and concentric Glass-patterns have shown that adaptation is global-form specific, but transferable between color and luminance information, further suggesting a common form system combining both luminance and color information (Rentzeperis and Kiper, 2010). In this study, luminance adaptation was found to have a stronger effect in general, and there was also some color-specificity in color adaptation. However, results from other studies suggest that the global analysis stage in Glass pattern processing pools the signal across different color channels and has thus no color-selectivity (Clifford et al., 2004; Wilson and Switkes, 2005).

## Color, Form, and the Brain

The properties of visual perception, which are revealed by behavioral studies, are the result of the functioning characteristics of the visual brain. Therefore, a link between the neuronal and the behavioral levels of description is the ultimate goal in cognitive neuroscience. With respect to the relationship between color and form in particular, psychophysical results suggest that anatomical, physiological and imaging studies should address the following questions: (1) can neurons selective for the color of a stimulus also be selective for the orientation and SF of the stimulus, or are these mutually exclusive properties?, (2) are spatial and chromatic properties processed in topographically distinct parts of the brain?, (3) is it possible to drive spatially-selective neurons using stimuli defined by chromatic-contrast alone?, (4) if they exist, are these neurons color-signed or color-unsigned?, (5) are the tuning properties of these neurons similar to the ones driven by luminance?, (6) do luminance and isoluminant stimuli activate the same or different form-related areas in the brain, and how does that change as one moves along the hierarchy of the visual system? In this section, studies addressing these questions will be reviewed, in order to see whether and to what extent the neurobiology of the visual system reflects the picture emerging from the psychophysical evidence. The prediction of the latter is probably for monocular, orientation selective units formed within each of the two cardinal chromatic dimensions at the initial stage, either color-signed or color-unsigned, and neurons retaining sensitivity to color but lacking selectivity being produced by pooling of these initial signals at subsequent stages of spatial integration. However, although the link between physiology and behavior is perhaps the ultimate goal in the brain/mind sciences, it is not always a straightforward matter.

Years of electrophysiological and anatomical studies of the visual system in primates have revealed a cortical architecture which is mainly characterized by functional specialization (see Zeki, 1993): different visual attributes such as color, motion, and form are processed by separate neuronal populations, in different parts of the brain. The idea of such an architecture was initiated by studies in prestriate cortex, pointing toward a specialization for motion in area V5 (Zeki, 1974) and color in area V4 (Zeki, 1973). Segregation is present in the very early visual areas V1/V2 as well, in which different parts of the information are processed by separate neuronal populations, within different anatomical compartments (see Livingstone and Hubel, 1984; Shipp and Zeki, 1985, 2002; Moutoussis and Zeki, 2002). For example, cytochrome oxidase ‘blobs’ in V1 and ‘thin stripes’ in V2 contain chromatically tuned neurons that are indifferent to orientation and could thus support color perception. Anatomical studies nicely complement the physiology, showing that blobs project to thin stripes and the latter to V4, perhaps forming the color-from-color system referred to earlier. Similarly, the form-from-luminance system could be reflected in the interstripe-interblob-V4 pathway, containing orientation-selective neurons that are indifferent to the color of the stimulus. Perhaps Stephen Grossberg’s formulation is relevant here, regarding the pathway originating in blobs as a ‘feature contrast system,’ painting color into fields defined by the ‘boundary contrast system’ originating in the interblob pathway (Grossberg, 1994).

Several studies have challenged the robustness of such segregation, by demonstrating the presence of neurons with dual selectivities, or cells with selectivities which are different to the majority of the neurons in the particular anatomical region (e.g., Leventhal et al., 1995; Kiper et al., 1997; Friedman et al., 2003). In general, studies with extensive histological documentation of electrode tracks with respect to cytochrome stripes, report higher levels of stripe specialization than studies lacking such documentation (see Shipp and Zeki, 2002), and are further supported by optical imaging studies of V2 (Tootell et al., 2004; Chen et al., 2008; Lu and Roe, 2008; Lu et al., 2010; An et al., 2012). Hence, the overall picture of segregation retains credence, even if one chooses to take account of studies arguing for the opposite (see Gegenfurtner, 2003). Furthermore, the issue of V2 neurons with dual selectivities (reported by studies lacking any histological background) could be explained by the fact that such neurons are common in the feedback layers (that lack ascending output) but are less frequent in the ascending output layer of this area (Shipp et al., 2009).

Functional specialization is also in accord with psychophysical studies, showing that different visual attributes are being perceived at different times (Moutoussis and Zeki, 1997a,b), and is the general principle by which visual processing is implemented by the brain (for an alternative view see Lennie, 1998). It should be noted, however, that most of the conclusion regarding the functional architecture of the visual cortex are based on the assumption that individual neurons function as independent ‘feature-detectors.’ In this way, brain regions are thought to contain neurons with similar tuning characteristics, and therefore the function of the area as a whole is fully described by the function of individual neurons. In more recent years, the idea that information is encoded in the *pattern* of neuronal activation has prevailed, making the interpretation of individual-neuron-selectivities less straight-forward (see Rigotti et al., 2013 for one nice example).

With respect to color and form in particular, many electrophysiological studies have concentrated on showing that orientation selective cells are not selective for the color of the stimulus and vice versa (see above). However, most of these studies use luminance contrast to test for orientation selectivity and simply verify the fact that the form-from-luminance system is separate from the color-from-color system, which carries the information regarding the wavelength composition of the light. As discussed in previous sections, the latter is used to determine the color of the various objects, but could also be used to inform form perception. In searching for the correlates of such a chromaticity-based form system, one should look for spatially-selective information, using stimuli defined by color contrast alone. It is important to note that, although color-signed neurons are necessary for encoding the color of an object, form-from-color can be encoded by color-unsigned responses – i.e., responses to pure chromatic boundaries without any selectivity for the particular configuration of colors either side of a contour.

Along these lines, orientation-selective neurons responding to pure color stimuli have been reported in area V1, and these cells were also found to be tuned to various spatial frequencies (Thorell et al., 1984). Most of these neurons were color-tuned and responded to luminance modulation as well, although it is possible that multi-unit recording might have contaminated such early electrophysiological results. This is the first study to describe a color-unsigned, orientation specific response. These were complex cells that were found to respond to multiple colors (though not defined within DKL space). Color-signed simple cells were also reported. Although eye-preference was tested for each neuron, no further information is given regarding the monocularity or not of the various cell types. Compared to the luminance-cells, color-cells were found to be more broad-band, with a higher peak at low spatial frequencies but equally sensitive to luminance-cells at high frequencies. The presence of multiple orientation and SF channels for color information makes a Fourier-type analysis of spatial chromatic-information possible, similar to that proposed for luminance (Campbell and Robson, 1968). These results differ from the ones in the LGN, which is low pass for color and high pass for luminance, and where the P system has been shown to respond to isoluminant stimuli much better than the M system (Hubel and Livingstone, 1990). Pure-color responses in primary visual cortex were also reported in a subsequent study, in which the more strongly color-tuned responses were at the same time unselective for the orientation of the stimulus (Lennie et al., 1990). Color-signed responses were found in this study, but the responses of the majority of the complex cells were independent of the chromaticity of the stimulus used to measure them, as in Thorell et al. (1984). It thus seems that, already at the stage of V1, spatial-vision mechanisms sensitive but not selective to color are already present. In yet another study in area V1 it was shown that color-selective cells as a population are tuned to lower spatial-frequencies than non-color cells (Leventhal et al., 1995). This study reports that most cells in layer 4Cβ also exhibit a significant degree of orientation bias. These cells are also monocular and parvo-driven (hence R/G channel selective), and thus provide a possible neuronal substrate for the signed, monocular oriented mechanism implied by psychophysics.

Color-selective cells responding to isoluminant stimuli have been also reported in area V2 (Kiper et al., 1997). For most of these cells, the addition of luminance contrast was found to increase the strength of the responses, but their orientation and spatial-frequency tuning was similar when using either luminance or chromatic stimuli. They found many more color-unsigned than color-signed cells. The former can be equated with the majority of units showing linear cone summation, and which were noted to respond equally well to light red/dark green gratings or dark red/light green. The minority units with non-linear, narrower cone summation did not show this property, but were phase selective for the combination of the chromatic and achromatic components of the gratings (Kiper et al., 1997). In agreement with the potential of chromatic information to support form perception, cells responding to chromatic edges, with or without selectivity to contrast polarity, have been reported both in V1 and in V2 (Friedman et al., 2003). Cells responding to uniform color-fields (vs. color edges) were also reported, suggesting a role for these areas in the perception of the internal color of a surface (vs. contour signaling). The latter is in agreement with results from human fMRI experiments, showing that neon-color filling-in can selectively activate striate cortex (Sasaki and Watanabe, 2004). Finally, form-from-color can alter the responses of neurons in striate cortex in the same way that form-from-luminance (or texture, motion and depth) can: a robust contextual modulation in V1 responses by figure-segregation has been reported, the latter being purely defined by color-contrast well outside the receptive field boundary and thus implying spatial integration and feedback from higher areas (Zipser et al., 1996).

**FIGURE 3:**
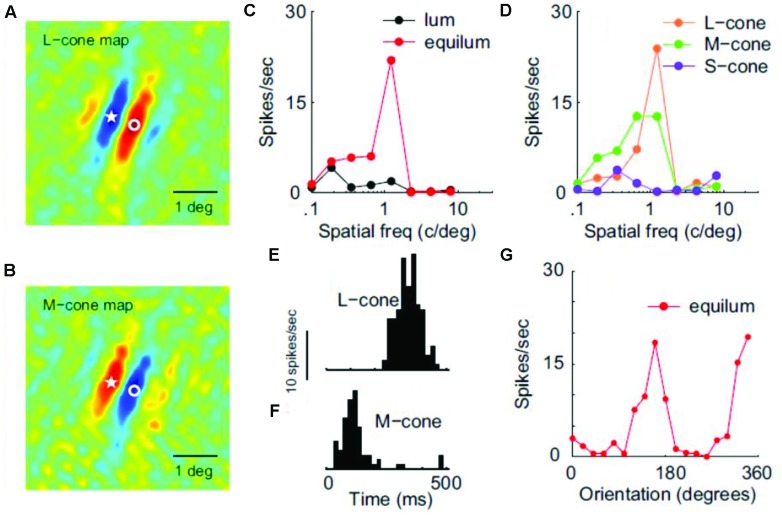
**Receptive filed properties of a double-opponent simple cell from area V1. (A,B)** Shows the spatial distribution of responses to L-cone and M-cone isolating patterns respectively (red: excitation, blue: inhibition). Star and circle represent corresponding point in the two images. **(C)** Shows SF tuning using luminance and chromatic stimuli. **(D)** Shows SF tuning for cone-isolating stimuli. **(E,F)** Shows temporal modulation by L-cone and M-cone isolating drifting gratings, revealing the opposite sign of the two cone inputs. **(G)** Shows the orientation tuning of this cell (Reprinted with permission from Society for Neuroscience; Johnson et al., 2008).

In a detailed electrophysiological study using luminance and isoluminant stimuli equated for cone-contrast, many V1 neurons were characterized as ‘color-luminance’ because they responded almost equally well to both (Johnson et al., 2001). Luminance cells, as well as a minority of color-only neurons (which were low-pass and unoriented), were also reported. In this study, the color tuning of neurons along different directions of color space was not examined in detail. Instead of comparing between different colors, the characterization of individual neurons was based on the ratio between the maximum isoluminant and luminance responses. A ‘color-cell,’ for example, does not necessarily have a particular color preference (i.e., it could be color-unsigned) but rather responds more strongly to pure-color gratings than to luminance ones. More importantly, most ‘color-luminance’ cells were tuned for SF and orientation in an equally selective way for chromatic and luminance patterns, revealing a spatial selectivity for color boundaries in primary visual cortex and thus making them a good candidate as the neural correlate of the ‘form-from-color’ system. In a subsequent study by the same group, these ‘color-luminance’ cells were renamed as ‘double-opponent’ and were found to consist 30% of the total V1 population (Johnson et al., 2008). They are color-signed, i.e., have a simple type of receptive field that retains color-sign, and take their name from the presence of both spatial and cone opponency (see **Figure 3**). Double-opponent neurons (whether orientation selective or not) could potentially signal the local light-composition comparisons (see Land’s Retinex algorithm above), and thus be the building blocks of color constancy calculations (color-from-color). Orientation-selective double-opponent neurons that respond to pure chromatic patterns, could also be part of the form-from-color system in striate cortex (Johnson et al., 2008; Conway, 2009). Yet another 10% of the population was found to be single-opponent, non-selective for orientation, low-pass and not responding well to luminance, making them a good candidate for signaling the wavelength composition coming from a region. Finally, the remaining 60% of the V1 population was found to be non-opponent; these cells were also the most orientation selective, making them a good candidate for the form-from-luminance system.

The prescription arising from the survey of the psychophysical literature is for color-tuned, oriented units to be monocular (or, at least, to display marked ocular dominance). Unfortunately, the studies described above provide scant information on this topic. Earlier electrophysiological and optical imaging studies do indeed indicate that cytochrome oxidase blobs represent monocular sites of color processing in primate striate cortex (Horton and Hubel, 1981; Livingstone and Hubel, 1984; Tootell et al., 1988; Ts’o and Gilbert, 1988; Ts’o et al., 1990; Landisman and Ts’o, 2002; Lu and Roe, 2008). But, problematically, their consensus is that the monocularly-driven color-processing cells lack selectivity for orientation, in apparent conflict with the behavioral results. This conflict may be eased by noting that the degree of orientation tuning observed can depend on the stimulus used: low frequency sinusoidal gratings characteristically elicit tighter tuning than rectangular bars (Leventhal et al., 1995). Indeed, most of the studies mentioned above that investigate the relationship between cytochrome oxidase blobs and orientation tuning, have used single bars as stimuli, with the exception of Landisman and Ts’o (2002) and Lu and Roe (2008): the latter two studies have used gratings instead, and report a less strict separation between blobs and orientation tuning. Furthermore, a more recent study using (achromatic) sinusoidal gratings reported appreciable orientation tuning in blobs that was only marginally inferior to that observed in interblobs – an orientation bandwidth of 28.4° for blobs and 25.8° for interblobs (Economides et al., 2011). This tantalizing picture of the nature of early physiological mechanisms vis-à-vis psychophysical inference requires clarification; ideally perhaps, an analysis of the trial-by-trial psycho-physiological correlate in monkeys trained to report subjective phenomena induced by a color-contingent tilt -illusion paradigm.

Moving from the early visual areas to prestriate cortex and thus higher in the hierarchy of visual processing, the presence of color-selectivity in V4 has been a seminal finding toward the idea of functional specialization in the visual system (Zeki, 1973). Furthermore, some cells in this area seem to encode the perceived color rather than the local wavelength-composition of the stimulus (Zeki, 1983; Kusunoki et al., 2006). The presence of a similar ‘color-center’ has been also reported in the human (Lueck et al., 1989; Bartels and Zeki, 2000) and, although many visual areas can decode the color of a stimulus using multivariate fMRI analysis, a color-space representing the perceptual organization of colors can only be found in V4 (Brouwer and Heeger, 2009). Anatomically, segregation and clustering of color-selective neurons has been reported in V4, similar to the cortical architecture found in areas V1 and V2 (Yoshioka and Dow, 1996). A picture of compartmentalization also emerges from a study combining monkey fMRI and electrophysiological recordings in V4 and more anterior areas in the inferotemporal cortex, revealing color-selective compartments named ‘globs,’ in which cells also show some shape selectivity but not as strong as in the ‘interglobs’ (Conway et al., 2007).

With respect to the color-form relationship, some neurons in area V4 have been reported to carry signals encoding feature contrast in either shape or color (Ogawa and Komatsu, 2004). In the same study in alert, task-trained subjects, the responses of these neurons could be modulated by top-down attention to a particular type of singleton. In another electrophysiological study, 22% of the V4 neurons showed the color-unsigned property of responding maximally at isoluminance without necessarily being color selective, but could also show form-selectivity when tested with various different shapes (Bushnell et al., 2011). In a subsequent study from the same group, the shape selectivity of V4 neurons was found to be the same when tested with two different colors – confirming the presence, in this higher visual area, of mechanisms which are color-sensitive but not color selective (Bushnell and Pasupathy, 2012). There is thus a selectivity to form defined purely from color contrast in V4, irrespective of the particular color that is being used. In this study, 35 V4 neurons were tested with different colors and shapes and the selectivities for these two attributes were found to be independent from each other.

Similar results have been reported in area IT, where again the selectivity to color (tested with non-isoluminant stimuli) was found to be independent from the selectivity to form, with color selectivity remaining the same across different shapes (Komatsu and Ideura, 1993). Independence between color and shape preference has been also reported in a study in which IT neurons were tested using combinations of two shapes and two colors (McMahon and Olson, 2009). For most neurons in this study, responses were a linear sum of their color and shape preference, indicating independent coding of the two, with only a few neurons showing some interaction effects and thus being suitable for coding conjunctions. It is interesting to note that the conjunction-responses were no later than responses to single features, implying no extra time for feature binding (McMahon and Olson, 2009).

A functional segregation between color, faces and form, with at least three representations of the visual field, has been revealed in area IT using fMRI both in human (see Bartels and Zeki, 2000) and monkeys (Lafer-Sousa and Conway, 2013). In the latter study, the more pronounced distinction was found between color and faces, with color regions weakly responding to non-face forms and with optimal form responses outside color regions. The authors suggest that the form-selectivity found in color regions could perhaps be attributed to the second, color-based form system (form-from-color). The argument is not very convincing, however, since what is meant by ‘form-selectivity’ is a weakly differentiated response between fruit, bodies and places. Furthermore, the form-from-color system is not necessarily color-selective and would thus not show up in an fMRI cognitive-subtraction type of experiment. It is more likely that these color regions in IT are part of the color-from-color system, initiated in the V1 blobs and proceeding via the V2 thin stripes and V4 globs (see above).

The neural-basis of interactions between color and form has been also studied in humans, using fMRI. In one such study, subjects were adapted to either one of two different orientations defined by color (reddish/greenish) or luminance (four conditions in total), and the weakest rebound-response (i.e., the response following adaptation) in areas V1–V4 was found when the test was the same orientation and chromaticity (chromatic or achromatic) as the adaptor (Engel, 2005). Some transfer was observed between color stimuli of a different orientation, suggesting a pure color-adaptation, and not much transfer between luminance and color stimuli of the same orientation, suggesting separate populations and thus an independent chromatic input to form perception (Engel, 2005). Additionally, an ANOVA model was used in this study to show that joint adaptation effects can be greater than the sum of color-only and orientation-only effects, suggesting the existence of a neuronal population which is jointly selective for both color and orientation. It is not clear from the design of this experiment whether color-selective neuronal mechanisms are necessarily involved, as color-unsigned neurons could also account for the effect.

Although adaptation and rebound can be a valuable tool to overcome the limited spatial resolution of fMRI, caution should be taken before inferring selectivity in a particular area from such studies (for example, see Rentzeperis et al., 2012). Multivariate-analysis, looking at activation patterns instead of individual voxel responses, is a preferable technique for solving such problems. Using machine-learning type of analysis on human-fMRI data, statistical learning algorithms trained to distinguish between different color-orientation conjunctions have revealed the presence of color-form binding in early visual cortex (Seymour et al., 2010). Supporting a segregation of function, separate sets of voxels were found to best support conjunctions, orientation information or color information, although some voxel groups could also discriminate between their non-preferred stimuli (Seymour et al., 2010). With respect to color processing *per se*, V4 was found to have the best performance in color discrimination and was the only area to encode the latter more efficiently than orientation. In general, however, some caution is also necessary when inferring neuronal mechanisms from fMRI data, especially in the case of multivariate analysis where the strategy that the classifier uses for classification is not always clear.

Regarding the ability of chromatic information to support form perception, another human fMRI decoding study has shown that areas V1–V3 can perform orientation discrimination using luminance, M-L cone, and S-cone stimuli (Sumner et al., 2008). Furthermore, since no transfer was found between conditions (e.g., training the algorithm with luminance and testing with M-L stimuli) this finding suggests the presence of at least three separate orientation-selective neuronal populations in each of these areas, responsive to particular directions of color space. Although it is not always wise to extrapolate fMRI data to the level of single cells, this color specificity suggests the involvement of color-selective neuronal populations in form processing. These results were confirmed by another human fMRI study, in which there was an orientation-specific response-reduction to a target stimulus (when surround and target had the same orientation), both for luminance and isoluminant gratings in areas V1, V2, V3, V3A, and V4 (McDonald et al., 2010). A weak cross-effect reduction was found in some areas, suggesting once more the existence of separate luminance and chromatic orientation-selective neuronal populations, but also the presence of orientation mechanisms which are indifferent to the nature of the signal. Finally, prestriate areas (but not V1) have been shown to code for complex spatial structure (phase congruency) in a human fMRI study using isoluminant stimuli, suggesting again that pure chromatic information can be used in higher order form-vision calculations (Castaldi et al., 2013). Furthermore, by using even- and odd-symmetric isoluminant phase-congruent stimuli, this study implies the presence of color-signed mechanisms throughout visual cortex.

## Conclusion

Information about the wavelength composition of light reflected by the various parts of the visual field can be used twofold: in order to compute color and attribute it to the various objects in the scene, and in order to detect chromatic boundaries and see shapes and forms. The latter can also be the result of processing luminance information, which is not necessarily redundant with that provided by the chromatic system. Furthermore, in addition to the ability of chromatic cues to support form perception, color *per se* can also influence the perception of form, at various stages of the processing hierarchy.

A contingency between form and color has been revealed from several psychophysical experiments, using adaptation and illusions. Such contingencies suggest that form information is coded independently by the color system, in a way that is equally effective to that of the luminance system. A coupling between color and form at early visual channels is further supported by the existence of dual-selective neurons at the early stages of visual processing, as well as by human fMRI data that reveal the presence of information regarding color-form conjunctions in early visual areas. Equality between form-from-luminance and form-from-color is evident in psychophysical studies comparing the spatial properties of color and luminance detectors, which reveal a similarity in the orientation and SF tuning characteristics of the two systems. The luminance system is more narrowly tuned and more sensitive at high SFs, whereas the color system performs better at low SFs and can in some cases dominate luminance information. Thus, psychophysical experiments investigating form detection at an early, local level, demonstrate the existence of equally efficient independent mechanisms for the detection of chromatic and luminance signals respectively, and the ability of chromatic information to support the early stages of form vision almost as effectively as luminance information. These separate mechanisms perform an independent early analysis of the spatial information of the image, but there also seems to be a certain level of interaction between them.

Overall, at a local level, the performance of the color system is not inferior to the performance of the luminance system and may sometimes be superior to it, making chromatic information just as valuable as luminance information in supporting form perception. A comparison between the efficiency of these different types of input has been also investigated in global form perception: at that stage, where the spatial integration of different local information takes place, the color system once more accomplishes global integration tasks in a way that is equally effective to that of luminance information. Interestingly, although some tasks reveal separate color and luminance integration systems with a limited amount of interaction between them, even at the global stage, others additionally suggest the existence of a single mechanism able to pool both types of information. Results from perceptual illusions also support this notion, showing independence between the different mechanisms at a monocular level but combination of color and luminance information at higher stages, after the input of the two eyes has been combined. It therefore seems that, at some point and depending on the task, the brain is able to bring together spatial information coming from different sources.

As far as the visual system is concerned, anatomical, electrophysiological, as well as imaging studies have shown that the mechanisms involved in perceiving color are separate and topographically segregated from those supporting the perception of form. Such architecture is in agreement with the general principle of a functional specialization in the visual brain. There are several studies supporting a functional segregation between color-from-color and form-from-luminance, although a possible segregation between the latter and form-from-color is not yet clear at the neurobiological level: some studies have even shown that both color-derived and luminance-derived form calculations can be performed by the same cells, in an equally effective way. More importantly though, our behavioral ability to perceive form defined by pure-color information is fully supported at the implementation level by the existence of spatial selectivity in neurons driven by isoluminant stimuli: cells responding to pure chromatic contrast are present in several visual areas and provide a good candidate for the color-derived form-vision.

Concluding, the relationship between color and form seems to be one of love, support and dependence, but also of separation, independency and, in some cases, rivalry. Color information can be carried by neurons which are either color-signed, i.e., selective for a particular color, or unsigned, i.e., are unselective but respond to pure color contrast. Electrophysiological studies have revealed the presence of both types of cells and results from both psychophysics and imaging suggest that both are necessary to explain various aspects of the form-color interaction.

## Conflict of Interest Statement

The author declares that the research was conducted in the absence of any commercial or financial relationships that could be construed as a potential conflict of interest.
